# Mixed Heavy Metal Exposure During Pregnancy Induces GDM-like Metabolic Dysfunction Associated with Glycer-Ophospholipid Metabolic Reprogramming and Altered Insig1 Expression: A Multi-Omics Study in Rats

**DOI:** 10.3390/toxics14040351

**Published:** 2026-04-21

**Authors:** Tianao Sun, Zhanyue Zheng, Yongjie Ma, Minglian Pan, Yingjie Zhou, Jingxia Wei, Xinyu Yuan, Jinhao Wan, You Li, Yan Sun

**Affiliations:** 1 School of Public Health, Guilin Medical University, Guilin 541199, China; 17791635922@163.com (T.S.); zhengzhanyue999@163.com (Z.Z.); mayongjie0606@163.com (Y.M.); pml18278456264@163.com (M.P.); 15221582965@163.com (Y.Z.); m18777893571@163.com (J.W.); 18285094298@163.com (X.Y.); wjhwio@163.com (J.W.); 2 Guangxi Key Laboratory of Environmental Exposomics and Entire Lifecycle Health, Guilin Medical University, Guilin 541199, China

**Keywords:** Pb, Cd, Mn, As, transcriptome sequencing, metabolomics

## Abstract

This study aimed to investigate whether mixed heavy metal exposure (lead, cadmium, manganese, and arsenic) during pregnancy induces gestational diabetes mellitus (GDM)-like phenotypes and to explore the associated molecular alterations. We examined the effects of exposure on metabolic disturbances using a Sprague-Dawley rat model exposed to low- and high-dose mixed heavy metals, with doses selected based on biomonitoring data. The results showed that high-dose mixed heavy metal exposure significantly increased blood glucose levels in rats, elevated the area under the curve (AUC) during the oral glucose tolerance test (OGTT), and induced insulin resistance and dyslipidemia. Concurrently, pathological examinations revealed hepatocyte steatosis, inflammatory cell infiltration, and mitochondrial abnormalities in liver tissues. Transcriptomic and metabolomic analyses identified significant disruption of the glycerophospholipid metabolic pathway following heavy metal exposure, suggesting the involvement of this pathway in the observed metabolic disturbances. Lasso regression analysis identified Insig1 as a candidate gene associated with lipid metabolic alterations, a finding subsequently validated by qPCR. Overall, mixed heavy metal exposure during pregnancy was associated with GDM-like metabolic abnormalities in rats. Disruption of glycerophospholipid metabolism and altered Insig1 expression likely contribute to these effects, providing molecular evidence linking mixed heavy metal exposure to gestational metabolic dysfunction.

## 1. Introduction

Gestational diabetes mellitus (GDM) has emerged as a major global public health challenge. According to the International Diabetes Federation, hyperglycemia in pregnancy was estimated to affect 19.7% of live births worldwide in 2024, with GDM accounting for approximately 79% of these cases [1]. Globally, GDM affects approximately 14% of pregnancies, with direct healthcare costs reaching $1.6 billion in the United States alone [2]. Women with a history of GDM are at a 7- to 10-fold increased risk of developing type 2 diabetes compared to those with normoglycemic pregnancies [3]. In China, GDM has become one of the most common pregnancy complications [4,5,6]. A recent meta-analysis reported a pooled prevalence of 15.6% under the IADPSG diagnostic criteria for studies conducted after 2010, with rural areas experiencing a faster rate of increase than urban areas [7]. These significant trends underscore the urgent need to identify modifiable environmental risk factors contributing to GDM pathogenesis. A key phenotypic hallmark of GDM is dysregulated glucose and lipid metabolism, which serves as the focus of the present study.

Heavy metals are major environmental pollutants and widely recognized health hazards. They accumulate in the environment through natural processes—such as weathering, volcanic activity, and forest fires—as well as anthropogenic activities including mining, smelting, fertilizer application, and fossil fuel combustion, which facilitate the widespread dispersion of toxic metals [8]. Human exposure occurs via multiple pathways, including food, air, water, and soil, posing substantial risks to public health [9]. Although metals are integral to biological systems and serve specific physiological functions [10], internal metal homeostasis must be tightly regulated, as both deficiency and excess can be detrimental. Deficiency of essential metals may lead to pathological conditions such as anemia, liver disorders, infertility, various syndromes, and even death [11]. Conversely, excessive heavy metal exposure can cause toxicity and has been linked to severe genotoxic and neurotoxic outcomes, including ataxia, encephalopathy, seizures, Parkinson’s disease, and Alzheimer’s disease [12,13]. Heavy metals have been increasingly recognized as significant risk factors for metabolic disorders [14]. Mechanistically, heavy metals disrupt glucose and lipid homeostasis through several interconnected pathways. Metal ions promote the generation of reactive oxygen species (ROS) via Fenton-type reactions and depletion of antioxidant defenses, leading to oxidative stress—a central driver of insulin resistance and β-cell dysfunction [15,16,17]. Heavy metals activate pro-inflammatory signaling cascades, including NF-κB and NLRP3 inflammasome pathways, resulting in elevated circulating cytokines such as TNF-α and IL-6, which impair insulin signaling in peripheral tissues [18,19]. Heavy metal exposure disrupts mitochondrial function and dynamics, compromising ATP production and promoting lipid accumulation in the liver and other metabolic tissues [20,21,22].

In Guangxi—one of China’s major non-ferrous metal production regions—extensive deposits of manganese, lead–zinc, and other metal ores have supported decades of intensive mining. Historically, outdated production technologies and inadequate pollution control measures have resulted in severe heavy metal contamination in certain areas, with the co-occurrence of multiple pollutants such as Mn, Cd, Pb, and As [23,24]. Lead (Pb) induces oxidative stress, disrupts calcium homeostasis, and impairs insulin signaling via ROS-mediated inhibition of key enzymes in the insulin signaling cascade [25,26]. Mechanistic studies have demonstrated that Pb exposure significantly increases reactive oxygen species (ROS) production in pancreatic β-cells, leading to impaired glucose-stimulated insulin secretion (GSIS), a defect that can be reversed by antioxidant treatment. Cadmium (Cd) promotes insulin resistance by activating inflammatory pathways and interfering with fatty acid synthesis, leading to hepatic steatosis and dyslipidemia [27,28,29]. Arsenic (As) inhibits insulin-stimulated glucose uptake by disrupting PI3K/Akt signaling and impairs pancreatic β-cell function through mitochondrial dysfunction and oxidative stress [30,31]. Manganese (Mn), in excess, causes mitochondrial dysfunction and oxidative damage to pancreatic β-cells, thereby reducing insulin secretion [32,33].

Despite the growing evidence linking individual heavy metal exposure to metabolic disorders, the vast majority of existing studies have focused on single-metal exposures, examining the effects of Pb, Cd, As, or Mn in isolation. However, real-world environmental exposure rarely involves a single pollutant; instead, populations are simultaneously exposed to complex mixtures of multiple heavy metals that may interact in synergistic, additive, or antagonistic ways. The exclusive focus on single metals in previous research therefore does not accurately reflect the complexity of real-world exposure conditions, potentially leading to underestimation or mischaracterization of actual health risks. To address this gap and accurately reflect real-world exposure conditions, the present study was designed to model a specific mixture of four heavy metals—Mn, Cd, Pb, and As—that are of particular concern due to their high pollution levels in Guangxi, a major non-ferrous metal production region in China. This mixture was formulated based on measured metal concentrations in a GDM population and subsequent conversion to animal-equivalent doses, thereby mimicking the complex co-exposure scenario typical of environmentally contaminated areas. Using Sprague–Dawley (SD) rats as an experimental model, we examined whether gestational exposure to this metal mixture, in comparison with individual metal exposures, induces GDM-like phenotypes. Furthermore, by integrating multi-omics approaches, we aimed to systematically elucidate the molecular mechanisms underlying GDM-related metabolic dysregulation induced by mixed heavy metal exposure. These findings are expected to provide a scientific basis for developing monitoring strategies and health risk prevention frameworks for heavy metal exposure in Guangxi and other similarly contaminated regions.

## 2. Materials and Methods

### 2.1. Animal Model and Experimental Design

Female adult Sprague–Dawley (SD) rats (8–10 weeks old) were purchased from Henan Skbios Biotechnology Co., Ltd. (Zhengzhou, China) and housed at the School of Public Health, Guilin Medical University under specific pathogen-free conditions (21–25 °C, 40–60% relative humidity, 12 h light/dark cycle), with ad libitum access to standard chow and water. After one week of acclimatization, all females were paired overnight (18:00–08:00) with males at a 1:1 ratio. Vaginal plugs were examined the following morning, and pregnancy was confirmed by vaginal smear analysis; this day was designated as gestational day 0 (GD0).

Pregnant rats were randomly divided into 3 groups: the blank control group, the low-dose Pb–Cd–Mn–As mixture group, and the high-dose Pb–Cd–Mn–As mixture group (*n* = 8 dams per group for phenotypic analyses). From GD2 to GD17, rats received the corresponding treatments once daily by oral gavage. Body weight gain and food intake were recorded daily throughout pregnancy.

### 2.2. Dose Selection and Administration

#### 2.2.1. Basis for Dose Selection

Dose levels were established using measured concentrations of Pb, Cd, Mn, and As in early-pregnancy urine samples collected from a GDM population in Guangxi, China, as reported in our prior research. The 95th percentile urinary concentrations for each metal were used as reference values representing high-end environmental exposure: Pb 12.5 μg/L, Cd 4.2 μg/L, Mn 8.6 μg/L, and As 35.4 μg/L. These values were used as biomonitoring-based reference concentrations to inform the selection of experimental doses for the animal model.

#### 2.2.2. Dose Conversion from Human to Animal Equivalent

The human urinary concentrations were converted to estimated blood levels via established toxicokinetic relationships, assuming steady-state distribution. The total blood metal burden was calculated assuming an average human blood volume of 5 L. The human burden (μg) was then converted to an equivalent rat dose using a human-to-rat conversion factor of 0.018, based on body surface area normalization following standard pharmacological principles. This conversion factor adjusts for metabolic rate differences between species and is widely accepted for extrapolating toxicological doses from humans to rodents.

#### 2.2.3. Application of Safety Factor

To obtain experimentally feasible doses for mechanistic evaluation, an additional scaling factor was applied to the rat-equivalent dose based on human biomonitoring-based reference values. This approach aimed to provide distinct exposure gradients for detecting metabolic and molecular changes in a sub-chronic gestational model. Consequently, the specific low-dose levels were established as follows: 16 mg/kg bw/day for Pb, 15 mg/kg bw/day for Cd, 12 mg/kg bw/day for Mn, and 0.5 mg/kg bw/day for As.

#### 2.2.4. High-Dose Determination

For each metal, the high-dose level was set at fivefold the low-dose level to establish a dose–response relationship and to assess potential threshold effects. This 5× difference provides sufficient separation between doses to detect graded responses while remaining within the range of subchronic toxicity without inducing overt maternal toxicity. The selected dose range was designed to ensure sufficient exposure contrast for evaluating dose-related phenotypic and molecular responses in the animal model.

#### 2.2.5. Final Dose Levels and Administration

Based on these calculations, the final dose levels for each metal are summarized in Table 1. In the mixture exposure group, Pb, Cd, Mn, and As were administered concurrently at their respective low- or high-dose levels. All metal salts were dissolved in ultrapure water, with a gavage volume of 10 mL/kg body weight. Treatments were administered once daily by oral gavage from gestational day (GD) 2 through GD 17.

### 2.3. Sample Collection

On GD 20, which was 3 days after the final metal dosing, dams were anesthetized with chloral hydrate (8% solution, 0.5 mL/100 g body weight), and blood was collected from the abdominal aorta. Animals were then euthanized by an overdose of chloral hydrate (20% solution) immediately after blood collection. Subsequently, various organs, tissues, and fluids (including liver and serum) were harvested. The uterus containing the placenta and fetuses was excised and weighed. All samples were immediately processed or stored at −80 °C until further analysis. This study was reviewed and approved by the Animal Ethics Committee of Guilin Medical University (Approval No: GLMU-IACUC-202510147).

### 2.4. Biochemical Assays

Pregnant rats received daily oral gavage of metal solutions from GD 2 through GD 17. On GD 18, 24 h after the final metal administration, animals were fasted for 12 h and then underwent an oral glucose tolerance test (OGTT). A 50% glucose solution was administered orally at 2 g/kg body weight. Blood samples were collected from the tail vein, and glucose concentrations were measured using a Logitech automatic glucometer at 0, 15, 30, 60, and 120 min following glucose administration.

On GD 20, blood was collected from the abdominal aorta of pregnant rats, clotted at 20 °C for 45 min, and then centrifuged to separate the serum (12,000 rpm, 4 °C, 10 min). Serum C-peptide levels were quantified using a commercial ELISA kit (Elirette, E-EL-R3004, Shanghai Keaibo Biotechnology Co., Ltd., Shanghai, China). Serum levels of HDL-C, LDL-C, triglycerides (TG), and total cholesterol (TCH) were measured using commercial kits (A112-1-1, A113-1-1, A110-1-1, A111-1-1) corresponding to each analyte, respectively, following the manufacturer’s instructions.

### 2.5. Histopathology and Transmission Electron Microscopy (TEM)

#### 2.5.1. Histopathological Analysis

Maternal liver tissues were fixed in 4% paraformaldehyde for 24 h, dehydrated with graded ethanol, embedded in paraffin, sectioned at 5 μm, and stained with hematoxylin and eosin (H&E). Histological observation and image acquisition were performed using a bright-field light microscope (Zeiss, Oberkochen, Germany). Where applicable, histological assessments were performed by investigators blinded to group allocation.

#### 2.5.2. Transmission Electron Microscopy (TEM)

Small pieces (~1 mm^3^) of maternal liver tissue were immediately immersed in 2.5% glutaraldehyde fixative. Samples were then processed following standard procedures, including post-fixation, graded dehydration, infiltration and embedding, polymerization, ultrathin sectioning, and staining. Sections were examined with a transmission electron microscope for image acquisition and ultrastructural analysis, with a focus on mitochondrial morphology.

### 2.6. Omics Analyses

#### 2.6.1. Transcriptomic Analysis

For transcriptomic analysis, four pregnant rats were randomly selected from each of the blank control group and the high-dose Pb–Cd–Mn–As mixture group. Total RNA was extracted from maternal liver samples using TRIzol reagent according to the manufacturer’s protocol. RNA integrity and concentration were assessed using an Agilent 2100 Bioanalyzer. mRNA was isolated, and cDNA libraries were constructed following the standard Illumina protocol; subsequently, sequencing was performed on an Illumina HiSeq™ platform (San Diego, CA, USA).

Raw reads were quality-filtered to obtain clean reads, which were then aligned to the reference genome. Differential expression analysis was conducted using DESeq2 with raw read counts as input. Differentially expressed genes (DEGs) were defined as those exhibiting |log_2_(fold change)| ≥ 1 and an adjusted *p*-value < 0.05. Functional enrichment analyses of DEGs were performed using Kyoto Encyclopedia of Genes and Genomes (KEGG) pathways and Gene Ontology (GO) annotations.

#### 2.6.2. Quantitative PCR Validation

To validate transcriptomic profiles, quantitative real-time PCR (qRT-PCR) was performed using selected DEGs. Total RNA was extracted from maternal liver tissue using TRIzol reagent (Invitrogen, Carlsbad, CA, USA). Subsequently, 1 μg of total RNA was used for cDNA synthesis. qRT-PCR was conducted using the PrimeScript™ RT reagent kit with gDNA Eraser (Accurate Biology, Changsha, China) on a StepOne Plus Real-Time PCR System (Applied Biosystems Thermo Fisher Scientific, Shanghai, China). Gapdh was used as the reference gene, and amplification specificity was confirmed by melt-curve analysis at the end of each PCR run. Relative gene expression levels were calculated using the 2^−ΔΔCt^ method. Primer sequences are listed in the support information.

#### 2.6.3. Metabolomic Analysis

For metabolomic analysis, six maternal liver samples were randomly selected from the blank control group and the high-dose Pb–Cd–Mn–As mixture group and subjected to untargeted metabolomic profiling via ultra-performance liquid chromatography coupled with mass spectrometry (UPLC–MS, Thermo Fisher Scientific, Shanghai, China). Briefly, 50 mg of each liver sample was homogenized in 1000 μL of internal standard extraction solution (1000:2, *v*/*v*) via vortexing for 30 s, followed by sonication in an ice–water bath for 5 min. The mixtures were then extracted with a methanol–water mixture (1:1, *v*/*v*), vortexed, centrifuged, and filtered to obtain the supernatant for metabolite analysis. The UPLC–MS system was operated in both positive and negative ionization modes to acquire metabolomic features defined by unique mass-to-charge ratios and retention times.

Raw data were processed using Progenesis QI software 2.0 (Nonlinear Dynamics, Durham, NC, USA), with peak deconvolution performed under default parameters. Metabolite identification was conducted using a two-step strategy based on MS^1^ and MS^2^ spectra. First, an in-house metabolite library (Beijing Biomarker Technologies Co., Ltd., Beijing, China), containing chemical standards and manually curated compound lists, facilitated preliminary identification through the matching of accurate mass (*m*/*z*, ±5 ppm), retention time, and spectral patterns. Second, public databases—including HMDB, PubChem, METLIN, and KEGG—were consulted to further refine and confirm metabolite identities based on accurate mass, isotopic patterns, and MS/MS spectra. This workflow yielded metabolites identified at Metabolomics Standards Initiative (MSI) levels 1 or 2, with theoretical fragment matching and mass deviations within 10 ppm. All identified fragments were additionally verified using an in-house scoring system.

### 2.7. Statistical Analysis

Data normality was assessed using the Shapiro–Wilk test, and homogeneity of variances was evaluated using Levene’s test. Comparisons among multiple groups were performed using one-way analysis of variance (ANOVA). Based on the results of Levene’s test, when variances were homogeneous, Tukey’s honestly significant difference (HSD) test was applied for all pairwise comparisons; when variances were heterogeneous, the Games-Howell post hoc test was used, as it does not assume equal variances. For two-group comparisons, the two-tailed Student’s *t*-test was used, with Welch’s correction applied when variances were unequal. For omics data, *p*-values were adjusted using the Benjamini–Hochberg false discovery rate (FDR) method, with q-values < 0.05 considered statistically significant. All analyses were performed using SPSS (version 22.0) and R software (version 3.4.1). A two-sided significance level of α = 0.05 was applied for all hypothesis tests. To identify key genes associated with metabolic disturbances induced by mixed heavy metal exposure, least absolute shrinkage and selection operator (Lasso) regression was applied to the transcriptomic data. Lasso regression is a regularization technique that performs variable selection by shrinking the coefficients of less important features to zero, thereby identifying the most relevant predictors. The analysis was performed using the glmnet package in R software, with the optimal regularization parameter (λ) selected via 10-fold cross-validation based on the minimum mean cross-validated error. Genes with non-zero coefficients in the Lasso model were considered candidate key genes.

## 3. Results

### 3.1. Mixed Heavy Metal Exposure Induces GDM-like Phenotypes and Hepatic Pathology

**Figure 1:**
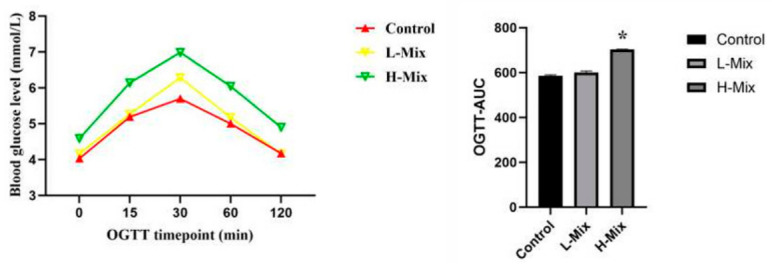
Effects of gestational mixed heavy metal exposure on maternal glucose tolerance in rats. Data in the line and bar plots are presented as mean ± standard error (SEM). “*”: *p* < 0.05.

**Figure 2:**
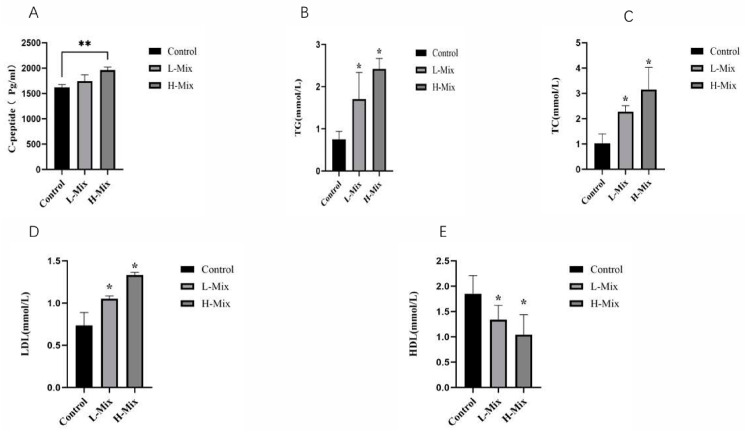
Effects of Heavy Metal Exposure on Glucose Metabolism and Lipid Profiles in Maternal Rats. (**A**) Effects of Heavy Metal Exposure on c-peptidein Maternal Rats. (**B**) Effects of Heavy Metal Exposure on triglycerides Maternal Rats. (**C**) Effects of Heavy Metal Exposure on total cholesterol Maternal Rats. (**D**) Effects of Heavy Metal Exposure on low-density lipoprotein Maternal Rats. (**E**) Effects of Heavy Metal Exposure on high-density lipoprotein Maternal Rats. The height of the bars represents the mean levels in each group, with the error bars indicating the standard error. “*”: *p* < 0.05, “**”: *p* < 0.01.

To determine whether gestational exposure to mixed heavy metals induces GDM-like metabolic dysfunction, we first assessed glucose and lipid homeostasis in pregnant rats. The oral glucose tolerance test (OGTT) revealed that rats in the high-dose mixed metal exposure group exhibited significantly elevated blood glucose levels at 30 min post-glucose administration compared to controls, with a corresponding increase in the area under the curve (AUC) (Figure 1). Consistent with impaired glucose tolerance, serum C-peptide levels were significantly elevated in the high-dose mixture group, indicating compensatory hyperinsulinemia—a hallmark of insulin resistance (Figure 2). In addition, lipid profiling showed significant increases in serum triglycerides (TG), total cholesterol (TC), and low-density lipoprotein (LDL), concomitant with a decrease in high-density lipoprotein (HDL), in the high-dose mixture group relative to controls (Figure 2). These findings demonstrate that high-dose mixed metal exposure disrupts both glucose and lipid metabolism, recapitulating key features of GDM.

**Figure 3:**
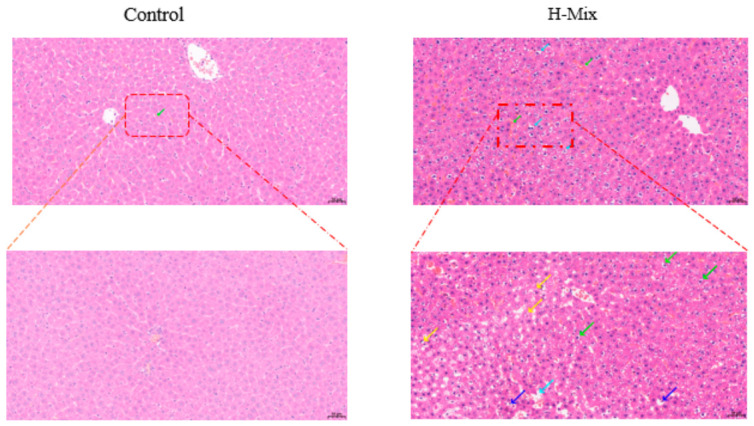
Effects of Mixed Heavy Metal Exposure During Gestation on Liver Pathology in Pregnant Rats: Pathological Changes Assessed by Hematoxylin and Eosin (H&E) Staining. In the H-Mix group, the central vein is located in the center of the hepatic lobule. Numerous hepatocytes show edema (green arrows), with swollen cells and loose, pale cytoplasm. Significant hepatic steatosis is observed (yellow arrows), and tiny round vacuoles are visible within the cytoplasm. Occasional nuclear displacement is noted, along with small-scale dilation of the hepatic sinusoids (drak blue arrows), widened septa, and loose, disorganized arrangement of hepatocytes; Scattered lymphocytic infiltration is occasionally seen within the parenchyma and hepatic sinuses (light blue arrows).

**Figure 4:**
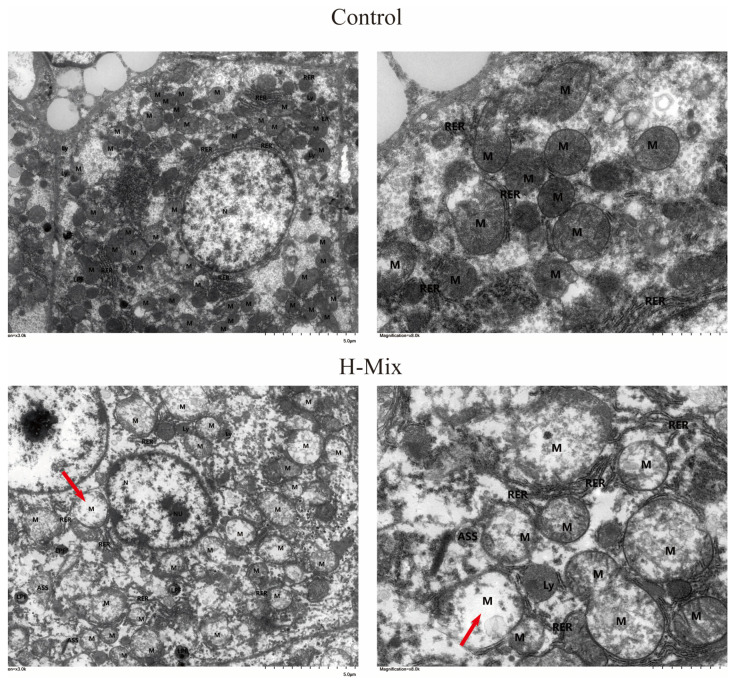
Transmission Electron Microscopy (TEM) Results. In the H-Mix group, hepatocytes exhibit moderate cytoplasmic edema with extensive areas of low-electron-density edema within the cytoplasm; organelles are markedly swollen. The nuclei (N) are oval in shape, with a high degree of dissolution of the chromatin; the nuclear envelope structure is blurred, and no significant widening of the perinuclear space is observed; Mitochondria (M) are abundant, mostly showing moderate to severe swelling; the matrix distribution is uneven; cristae are markedly fractured or absent; some membrane structures show localized dissolution or rupture; isolated mitochondria exhibit focal vacuolization (indicated by red arrows); The rough endoplasmic reticulum (RER) shows no significant dilation, with abundant ribosomes on its surface; a small amount of lipofuscin (LPF), lysosomes (Ly), and autophagosomes (ASS) are visible in the cytoplasm.

Given that the liver is a central regulator of glucose and lipid homeostasis, we next examined hepatic histopathology. H&E staining revealed that hepatocytes in the high-dose mixture group were loosely and irregularly arranged, characterized by cellular swelling and numerous large lipid vacuoles, along with nuclear displacement and occasional inflammatory cell infiltration (Figure 3). In contrast, livers from the control group displayed a normal radial arrangement of hepatocytes around the central vein, with no evident pathological changes. Transmission electron microscopy (TEM) further revealed ultrastructural damage: mitochondria in the exposure group exhibited moderate-to-severe swelling, an unevenly distributed matrix, fragmented or absent cristae, and partial membrane dissolution (Figure 4). Notably, these mitochondrial abnormalities—particularly cristae disruption and membrane damage—are consistent with alterations in membrane lipid composition, indicating a potential association with disrupted lipid metabolism.

### 3.2. Transcriptomic and Metabolomic Analyses Reveal Glycerophospholipid Metabolism as a Central Pathway

To elucidate the molecular mechanisms underlying the observed metabolic and histopathological changes, we performed transcriptomic and metabolomic profiling of maternal livers.

**Figure 5:**
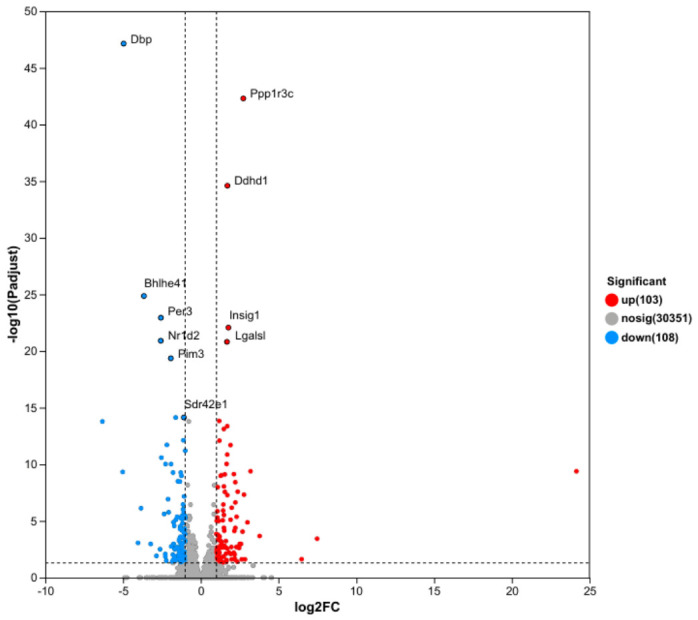
Volcano Plot of Differentially Expressed Genes.

**Figure 6:**
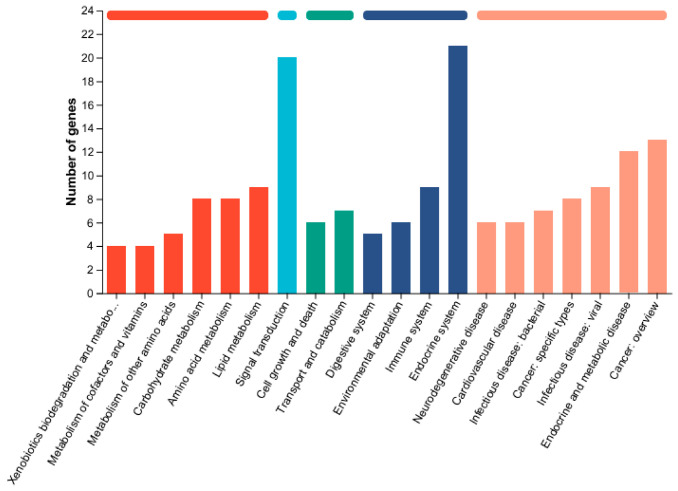
Functional enrichment analysis.

**Figure 7:**
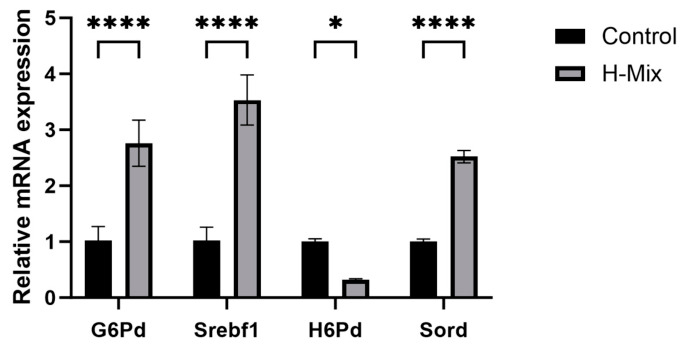
qPCR validation for the RNA-seq transcriptome in the liver of maternal rats (*n* = 4). Mean and standard error were reported. Relative expression level of each selected gene between heavy metal exposure group and control group were tested using nonparametric test. “*”: *p* < 0.05, “****”: *p* < 0.0001.

Transcriptomic alterations. RNA-seq analysis identified 211 differentially expressed genes (DEGs) in the high-dose mixture group compared to controls, with 103 upregulated and 108 downregulated (Figure 5). KEGG enrichment analysis revealed that mixed heavy metal exposure significantly altered multiple pathways, broadly categorized as metabolism (including carbohydrate, amino acid, lipid, and xenobiotic metabolism), cellular processes (such as signal transduction, cell growth and death, and transport), and disease-related pathways (including neurodegenerative, cardiovascular, and endocrine disorders) (Figure 6). Notably, lipid metabolism was among the most significantly enriched pathways, consistent with the observed dyslipidemia and hepatic steatosis (Figure 6). Specifically, genes involved in glycerophospholipid metabolism were among the most significantly altered. qPCR validation confirmed the expression trends of selected genes, corroborating the RNA-seq data (Figure 7).

**Figure 8:**
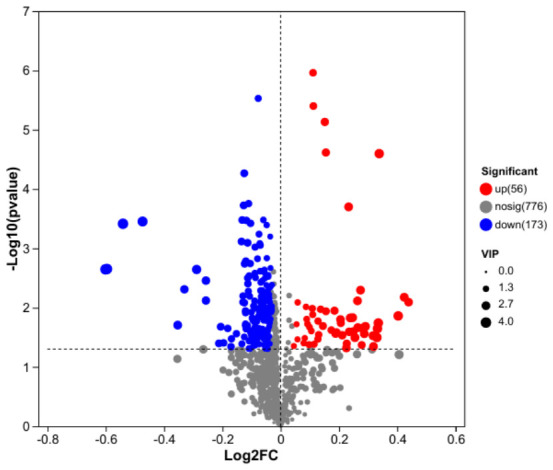
Volcano plot of differential metabolites between the high-dose mixture group and the control group.

**Figure 9:**
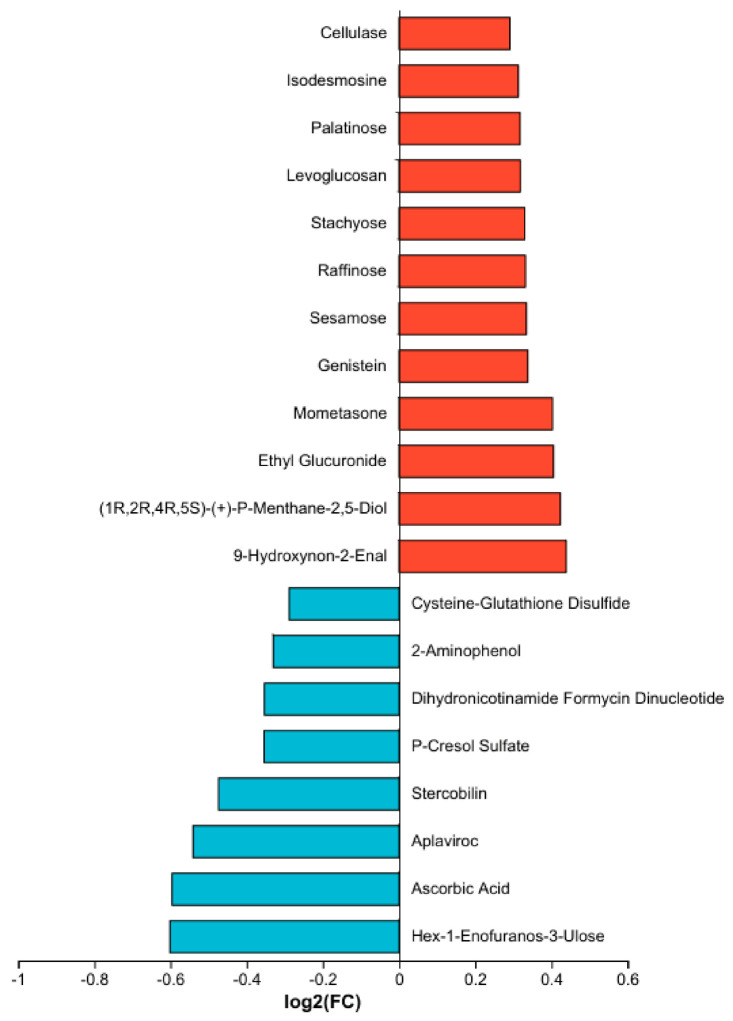
Classification of differential metabolites by functional category.

**Figure 10:**
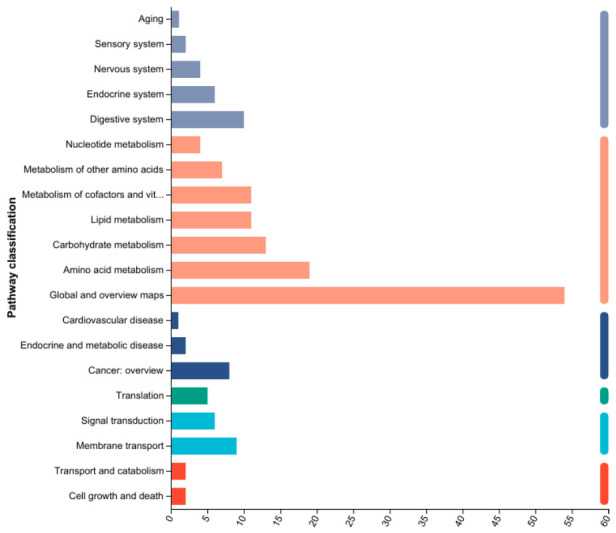
KEGG functional enrichment analysis.

Metabolomic alterations. Untargeted metabolomics identified 229 differentially abundant metabolites in the high-dose mixture group, of which 56 were upregulated, and 173 were downregulated (Figure 8). Antioxidant-related metabolites were predominantly downregulated. Specifically, levels of ascorbic acid (vitamin C) and cysteine-glutathione disulfide—a marker of glutathione redox status—were significantly decreased in the high-dose mixture group compared to controls. This pattern suggests a depletion of key antioxidant defenses. Carbohydrates and saccharides were predominantly upregulated. These included panose, palatinose, stachyose, raffinose, sesamose, levoglucosan, and sa-beta-gal. The accumulation of these saccharides may reflect disruptions in carbohydrate metabolism and glucose homeostasis. Lipid-related metabolites showed both upregulation and downregulation patterns. Notably, 9-hydroxynon-2-enal, a reactive aldehyde derived from lipid peroxidation, was significantly upregulated, indicating oxidative damage to membrane lipids. Other lipid-related species also exhibited altered abundance. Alterations were also observed in other functional categories, including folinic acid (a folate derivative), genistein (an isoflavone), and mometasone (a corticosteroid), suggesting broader metabolic reprogramming extending beyond glucose and lipid pathways (Figure 9). KEGG enrichment analysis revealed that differential metabolites were significantly enriched in pathways primarily related to metabolism (including lipid, carbohydrate, and amino acid metabolism), as well as in pathways associated with cellular processes and diseases (Figure 10). Full pathway details are provided in the corresponding figure and Supplementary Materials.

**Figure 11:**
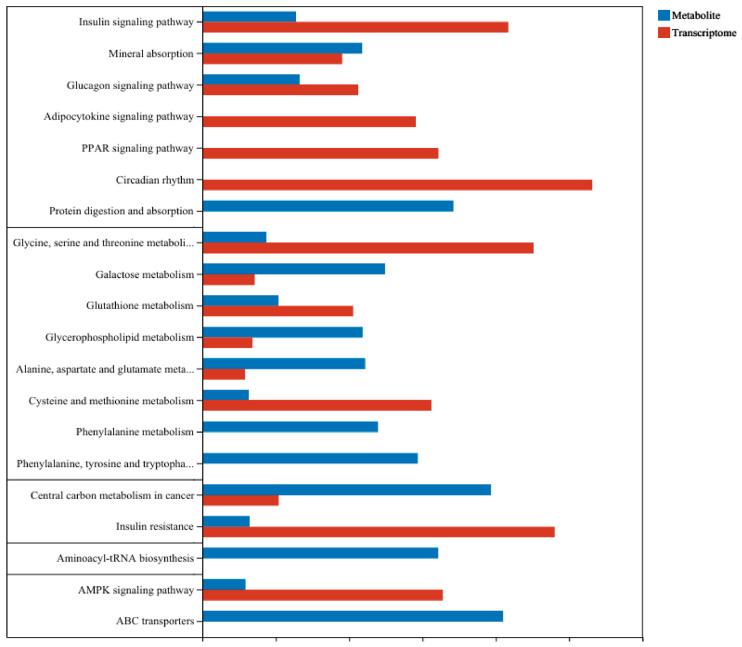
The number of DEGs and DMs involved in functional pathways.

Integration of multi-omics data. Joint KEGG enrichment analysis of transcriptomic and metabolomic datasets revealed glycerophospholipid metabolism as a co-enriched pathway (Figure 11), alongside other pathways including galactose metabolism, cysteine and methionine metabolism, and glutathione metabolism. Given that glycerophospholipids are essential components of cellular and mitochondrial membranes, the disruption of this pathway offers a mechanistic basis for the mitochondrial membrane damage observed by TEM. Furthermore, the concurrent downregulation of antioxidant metabolites (cysteine-glutathione disulfide, ascorbic acid) indicates the presence of oxidative stress, which may both contribute to and result from membrane lipid perturbations.

Co-enriched pathways. Joint KEGG enrichment analysis of transcriptomic and metabolomic datasets identified several pathways significantly altered at both the gene expression and metabolite levels. These included galactose metabolism, cysteine and methionine metabolism, alanine/aspartate/glutamate metabolism, glutathione metabolism, insulin resistance, glucagon signaling, and central carbon metabolism in cancer (Figure 11). Notably, glycerophospholipid metabolism emerged as the most consistently enriched pathway across both omics platforms, indicating its pivotal role in the metabolic response to mixed heavy metal exposure.

### 3.3. Insig1 Is Identified as a Candidate Gene Associated with Glycerophospholipid Metabolism and Phenotypic Changes

**Figure 12:**
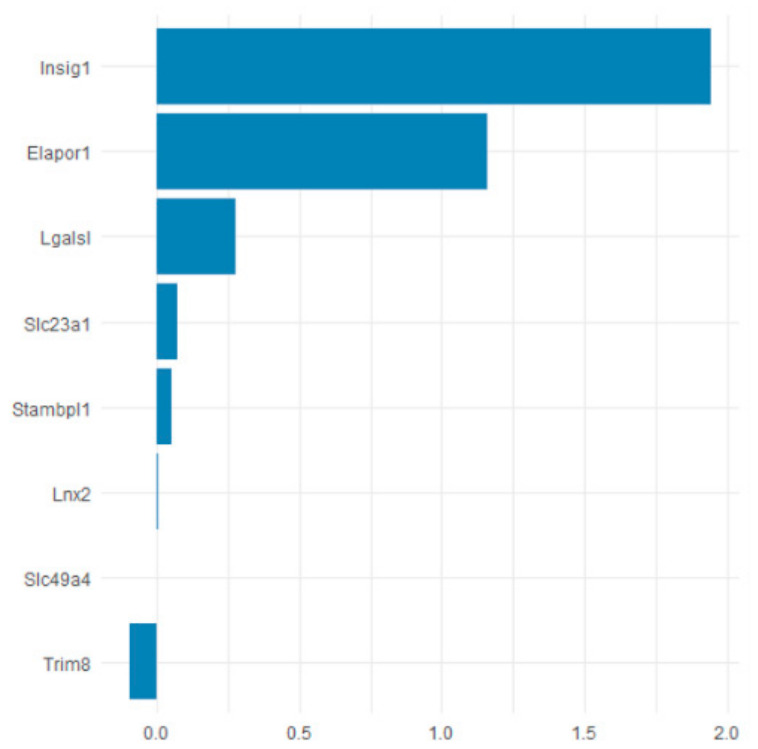
Results of Lasso Regression.

**Figure 13:**
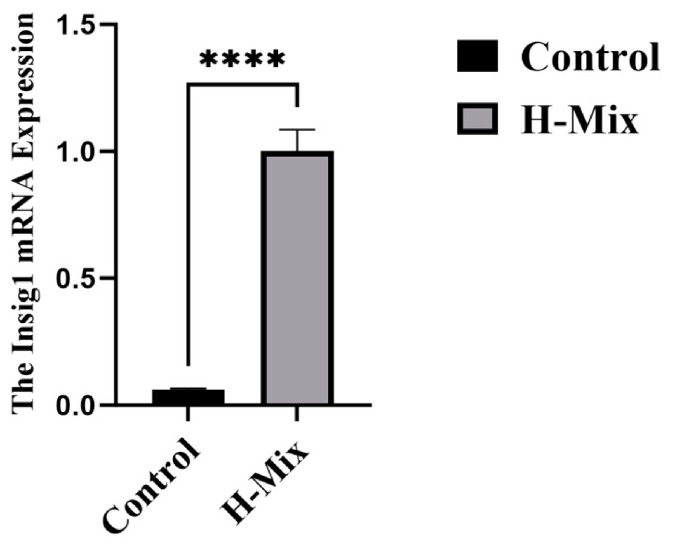
Expression Level of Insig1, “****”: *p* < 0.0001.

To prioritize genes linked to the observed metabolic disturbances, we performed Lasso regression on the transcriptomic data(Figure 12). This analysis highlighted Insig1 as a candidate gene associated with changes in glucose and lipid metabolism induced by metal exposure. Insig1 expression was significantly upregulated in the high-dose mixture group relative to controls, as confirmed by qPCR(Figure 13). Combined with the pathway enrichment results, these findings suggest that altered Insig1 expression may contribute to changes in glycerophospholipid metabolism and the observed metabolic phenotypes.

Insig1 is a well-established regulator of lipid homeostasis that acts by inhibiting SREBP processing and controlling cholesterol and fatty acid synthesis. The observed upregulation of Insig1 in response to metal exposure may represent a compensatory mechanism to limit excessive lipid synthesis; however, this adaptive response may also disrupt the normal balance of membrane phospholipids, contributing to the observed alterations in glycerophospholipid composition. Together with prior knowledge of Insig1 in SREBP signaling and lipid homeostasis, these findings provide a plausible molecular framework linking heavy metal exposure to altered glycerophospholipid metabolism and the metabolic and histopathological changes observed in this study.

## 4. Discussion

GDM is a complex metabolic disorder, and environmental pollution is considered a significant potential contributor to its increasing incidence. While existing studies suggest that single heavy metal exposure may disrupt glucose and lipid metabolism, the specific effects of multi-metal mixtures—commonly encountered in real-world environments—on metabolic health during pregnancy and their underlying molecular mechanisms remain inadequately understood. This study employed a rat model to simulate the typical environmental exposure scenario of Pb, Cd, Mn, and As mixtures in Guangxi, China. The results demonstrated that high-dose mixed heavy metal exposure during pregnancy induced GDM-like phenotypes in rats. Integrated transcriptomic and metabolomic analyses further suggested that dysregulation of glycerophospholipid metabolism is an important pathway associated with heavy metal-induced hepatic injury and systemic metabolic disturbance.

Insulin-induced gene 1 (Insig1) is a key regulator of lipid metabolism that plays a critical role in maintaining cellular lipid homeostasis [34,35]. Insig1 is an endoplasmic reticulum (ER) membrane protein that functions as a negative feedback regulator of sterol regulatory element-binding proteins (SREBPs), the master transcription factors controlling the expression of genes involved in cholesterol and fatty acid synthesis [36,37]. Emerging evidence suggests that Insig1 expression is altered in metabolic disorders such as non-alcoholic fatty liver disease (NAFLD) and type 2 diabetes, where it may contribute to hepatic insulin resistance and dyslipidemia [38,39].

This study provides experimental evidence that mixed heavy metal exposure during pregnancy is associated with GDM-like metabolic abnormalities in rats. Rats in the high-dose mixture exposure group (H-Mix) exhibited significantly elevated fasting and postprandial blood glucose levels, along with a marked increase in the area under the OGTT curve. Concurrently, abnormal elevations in serum triglyceride (TG), total cholesterol (TC), and low-density lipoprotein (LDL) levels were observed. Notably, serum C-peptide levels were significantly higher in the H-Mix group, indicating a state of compensatory hyperinsulinemia—an indicator of systemic insulin resistance (IR) [40,41].

The liver serves as the central hub for metabolic regulation, and its structural integrity is essential for maintaining glucose and lipid homeostasis [42]. Histopathological analysis revealed extensive lipid vacuolization and inflammatory infiltration in the hepatocytes of the exposed group. Crucially, transmission electron microscopy (TEM) results demonstrated significant damage to membrane systems: mitochondria exhibited swelling, cristae rupture, and the dissolution or disruption of membrane structures. The integrity of both mitochondrial and cellular membranes primarily depends on the stability of the phospholipid bilayer [43,44,45]. These ultrastructural pathological alterations provide a morphological basis for subsequent omics analyses and indicate a severe disruption in membrane lipid metabolism.

Integrated KEGG analysis of transcriptomics and metabolomics identified glycerophospholipid metabolism as one of the most significantly enriched pathways affected by mixed heavy metal exposure. This finding provides a novel perspective for understanding the pathogenesis of GDM. Glycerophospholipids are not only major components of cellular and mitochondrial membranes but also serve as lipid mediators in intracellular signaling [46,47,48]. First, the disturbance in glycerophospholipid metabolism provides a direct explanation for the mitochondrial membrane damage observed in this study. Heavy metals may interfere with the biosynthesis or remodeling of key phospholipids such as PC or PE, leading to altered mitochondrial membrane fluidity and permeability. This disrupts the electron transport chain and triggers oxidative stress [49,50,51]. In the current study, the significant downregulation of antioxidant metabolites like ascorbic acid and cysteine-glutathione disulfide supports the oxidative imbalance induced by membrane damage. Alterations in glycerophospholipid composition are closely associated with insulin resistance [52,53]. Insulin receptors are located within lipid rafts—membrane microdomains rich in specific glycerophospholipids and cholesterol [54]. Dysregulated glycerophospholipid metabolism can disrupt the lipid raft microenvironment, impairing insulin receptor phosphorylation and downstream PI3K/Akt signaling [55]. The concurrent enrichment of the “insulin resistance” and “PPAR signaling pathway” in our integrated analysis constructs a coherent evidence chain: heavy metal exposure → glycerophospholipid metabolic reprogramming → membrane (mitochondrial/cellular) damage → impaired insulin signal transduction → development of GDM.

The upregulation of Insig1 observed in this study is mechanistically linked to glycerophospholipid metabolism through its role in SREBP signaling. Insig1 is an ER membrane protein that functions as a negative feedback regulator of SREBPs, the master transcription factors controlling the expression of over 30 genes involved in cholesterol, fatty acid, and triglyceride synthesis [56,57]. Under sterol-sufficient conditions, Insig1 binds to SCAP, retaining the SCAP-SREBP complex in the ER and preventing SREBP activation [58]. The fatty acids synthesized under SREBP control—primarily palmitate and stearate—are essential building blocks for glycerophospholipid biosynthesis [59]. Moreover, SREBP-1c directly regulates GPAT3, a key enzyme in the initial step of glycerophospholipid synthesis. Sustained Insig1 upregulation, as observed in our study, may disrupt this feedback loop, leading to reduced nuclear SREBP levels and decreased expression of lipogenic genes [60]. Consequently, this leads to an altered supply of fatty acid precursors and reduced glycerophospholipid biosynthetic capacity, resulting in the glycerophospholipid metabolic reprogramming detected in our omics analyses. Given that glycerophospholipids are critical for mitochondrial membrane integrity, this disruption provides a mechanistic explanation for the mitochondrial damage observed by TEM, linking Insig1 dysregulation to the broader metabolic phenotype.

Furthermore, glycerophospholipids are essential components of cell membranes, and their metabolic intermediates, such as phosphatidic acid (PA), act as key precursors for triglyceride (TAG) synthesis. In the glycerolipid biosynthesis pathway, PA serves as an intermediate between the formation of phospholipids and diacylglycerol (DAG), with DAG serving as the direct precursor for TAG synthesis. This underscores the close relationship between glycerophospholipid metabolism and TAG biosynthesis. When the glycerophospholipid metabolic pathway is disrupted, the flow of intermediate metabolites may be redirected toward TAG synthesis, promoting lipid accumulation [61]. This mechanism explains the hepatic lipid deposition (steatosis-like changes) and dyslipidemia observed in this study.

In addition to its predominant role in lipid metabolism, this study also identified alterations in several amino acid metabolic pathways, including cysteine, methionine, alanine, and aspartate. Cysteine is a critical precursor for glutathione (GSH) synthesis, and GSH biosynthesis requires cysteine to react successively with glutamate and glycine via γ-glutamylation. As the most important low-molecular-weight intracellular antioxidant, GSH effectively scavenges reactive oxygen species (ROS) and maintains redox homeostasis. Therefore, disrupted cysteine metabolism results in decreased GSH levels, which is consistent with the reduced antioxidant capacity observed in metabolomics analyses [62]. These findings suggest that heavy metal-induced oxidative stress may further damage membrane phospholipids through lipid peroxidation, exacerbating glycerophospholipid metabolic disturbances and creating a vicious cycle of “oxidative damage–dysregulated lipid metabolism.”

In the high-dose mixed metal exposure group, we observed significant downregulation of key antioxidant metabolites, including ascorbic acid (vitamin C) and cysteine-glutathione disulfide [63,64]. Ascorbic acid is a primary water-soluble antioxidant that scavenges reactive oxygen species (ROS) and regenerates other antioxidants such as vitamin E. Cysteine-glutathione disulfide is an oxidized form of glutathione (GSH), and its accumulation reflects increased oxidative stress and depletion of the reduced GSH pool [65,66]. The reduction in these antioxidant metabolites indicates that the liver’s capacity to neutralize ROS was overwhelmed by metal-induced oxidative challenge [67]. Concurrent with antioxidant depletion, we observed significant upregulation of 9-hydroxynon-2-enal (9-HNE) in the high-dose mixture group. 9-HNE is a well-established biomarker of lipid peroxidation, generated during the peroxidation of ω-6 polyunsaturated fatty acids (PUFAs), particularly arachidonic acid and linoleic acid. 9-HNE is highly reactive and can form covalent adducts with proteins, impairing their function and contributing to cellular dysfunction [68,69]. The accumulation of 9-HNE provides direct molecular evidence that ROS generated in response to metal exposure have attacked membrane lipids, initiating lipid peroxidation chain reactions [70].

Based on our integrated multi-omics and phenotypic findings, we propose a mechanistic model linking mixed heavy metal exposure to GDM-like metabolic dysfunction. Lasso regression identified Insig1 upregulation as a key molecular event induced by metal exposure. Insig1 is a negative feedback regulator of SREBPs, which control lipid synthesis. Sustained Insig1 upregulation may disrupt SREBP processing, leading to dysregulated synthesis of glycerophospholipids—a pathway consistently enriched in our transcriptomic and metabolomic analyses. Glycerophospholipids are essential for mitochondrial membrane integrity; compromised glycerophospholipid homeostasis explains the mitochondrial damage observed by TEM, including swelling and cristae fragmentation. Mitochondrial dysfunction promotes oxidative stress (supported by downregulated antioxidants and upregulated lipid peroxidation products), culminating in the GDM-like phenotype (glucose intolerance, dyslipidemia, hepatic steatosis). This integrated model establishes Insig1 and glycerophospholipid metabolism as central nodes linking environmental metal exposure to gestational metabolic dysfunction.

Our findings are broadly consistent with epidemiological evidence linking prenatal or maternal exposure to heavy metals with abnormal glucose metabolism and increased GDM risk. However, whereas most previous studies have focused on individual metals, the present work addresses a mixed-exposure scenario that may better reflect environmental reality. In this rat model, mixed exposure to Pb, Cd, Mn, and As was associated with glucose intolerance, dyslipidemia, hepatic steatosis, and multi-omics alterations involving glycerophospholipid metabolism. These results support the importance of considering metal mixtures when evaluating metabolic health risks during pregnancy.

Several limitations should be acknowledged. First, this study was conducted in a rat model, and species differences may limit direct extrapolation to humans. Second, although dose selection was informed by human biomonitoring data, the experimental doses were scaled for mechanistic evaluation and may not directly represent typical environmental exposure levels. Third, the mixed-exposure design did not allow formal separation of additive, synergistic, or antagonistic effects among the component metals. Fourth, Insig1 was identified as a candidate gene through transcriptomic prioritization and qPCR validation, but functional experiments were not performed in the current study. Finally, the present analyses focused mainly on maternal liver and cross-sectional endpoints, without assessing other tissues, fetal outcomes, or long-term consequences. Future studies should address these issues through functional validation, broader tissue assessment, and longitudinal or interaction-focused designs.

## 5. Conclusions

This study comprehensively investigates the effects of mixed heavy metal exposure (Pb, Cd, Mn, and As) during pregnancy, demonstrating its potential to induce metabolic disturbances resembling GDM. In Sprague-Dawley rats, our results confirm that high-dose exposure to these metals leads to significant disruptions in glucose and lipid metabolism, as evidenced by impaired glucose tolerance, insulin resistance, and abnormal lipid profiles. Histological and ultrastructural analyses further revealed severe liver damage, including hepatocyte swelling, lipid vacuolization, and mitochondrial dysfunction, correlating with the observed metabolic changes. Integrated multi-omics analysis of transcriptomic and metabolomic data uncovered alterations in key metabolic pathways, particularly glycerophospholipid metabolism. This pathway’s disruption is central to the pathogenesis of GDM-like phenotypes induced by heavy metal exposure, providing novel molecular insights into the mechanisms underlying these disorders. Specifically, the upregulation of Insig1 was identified as a key molecular driver, influencing lipid metabolism and insulin resistance.

## Figures and Tables

**Table 1:** Summary of the final dose levels of each metal.

	Pb(CHsCOO)_2_	NaAsO_2_	MnCl	CdCl_2_
L-Mix	16 mg/kg bw/d	0.5 mg/kg bw/d	12 mg/kg bw/d	15 mg/kg bw/d
H-Mix	80 mg/kg bw/d	2.5 mg/kg bw/d	60 mg/kg bw/d	75 mg/kg bw/d

## Data Availability

Data will be made available on reasonable request.

## Supplementary Materials

The following supporting information can be downloaded at: https://www.mdpi.com/article/10.3390/toxics14040351/s1, Table S1: Sequence of PCR primer.
