# Supplementary material for: Mixed Heavy Metal Exposure During Pregnancy Induces GDM-like Metabolic Dysfunction Associated with Glycer-Ophospholipid Metabolic Reprogramming and Altered Insig1 Expression: A Multi-Omics Study in Rats
Source: Toxics. 2026 Apr 21;14(4):351. doi: 10.3390/toxics14040351 (PMC13120483; doi:10.3390/toxics14040351)
Supplement: Supplementary file 1 [file toxics-14-00351-s001.zip › toxics-4199729-supplementary.pdf]

Support Information

Table S1. Sequence of PCR primer

Primer Name	Primer sequence（5'-3')	Segment length（bp）
β-actin-F	GGCTGTATTCCCCTCCATCG	154
β-actin-R	CCAGTTGGTAACAATGCCATGT	
G6pd-F	CGAGGCCGTCACCAAGAAC	166
G6pd-R	GTAGTGGTCGATGCGGTAGA	
Srebf1-F	GCAGCCACCATCTAGCCTG	199
Srebf1-R	CAGCAGTGAGTCTGCCTTGAT	
H6pd-F	ATGAAGCACACAGGCATTTGG	170
H6pd-R	TCCAGGTATAGCTGAAACAGTCC	
Sord-F	GCTAAGGGCGAGAACCTGTC	151
Sord-R	CATGCTCCCAGTAGTGAACATC	
Insig1-F	CACGACCACGTCTGGAACTAT	213
Insig1-R	TGAGAAGAGCACTAGGCTCCG	

Effect sizes and confidence intervals

Outcome	Control	High-Dose Mixture	Mean Difference [95% CI]	Cohen's d
OGTT AUC	825.4 ± 45.2	1892.6 ± 82.5	1067.2 [982.3, 1152.1]	2.84
TG	0.8 ± 0.1	2.5 ± 0.3	1067.2 [982.3, 1152.1]	2.31
TC	1.6 ± 0.1	3.8 ± 0.4	2.2 [1.8, 2.6]	2.08
HDL-C	1.2 ± 0.1	0.6 ± 0.1	-0.6 [-0.7, -0.5]	2.45
LDL-C	0.4 ± 0.1	1.4 ± 0.2	1.0 [0.8, 1.2]	2.67
C-peptide	1.2 ± 0.1	3.4 ± 0.3	2.2 [1.9, 2.5]	2.78

Gene	log_2FC	95% CI for log_2FC	q-value	Effect size (η^2)
Insig1	1.42	[1.12, 1.72]	1.8×10^{-4}	0.52
Srebf1	1.18	[0.88, 1.48]	4.2×10^{-4}	0.48
Fasn	-0.92	[0.88, 1.48]	1.1×10^{-3}	0.39
Gpat1	-0.78	[-1.08, -0.48]	4.5×10^{-3}	0.31
